# Polyelectrolyte Multilayer Capsule (PEMC)-Based Scaffolds for Tissue Engineering

**DOI:** 10.3390/mi11090797

**Published:** 2020-08-22

**Authors:** Georgia Kastania, Jack Campbell, Jacob Mitford, Dmitry Volodkin

**Affiliations:** School of Science and Technology, Department of Chemistry and Forensics, Nottingham Trent University, Clifton Lane, Nottingham NG11 8NS, UK; georgia.kastania2016@my.ntu.ac.uk (G.K.); jack.campbell@ntu.ac.uk (J.C.); jacob.mitford2015@my.ntu.ac.uk (J.M.)

**Keywords:** CaCO_3_, growth factor, layer-by-layer, encapsulation

## Abstract

Tissue engineering (TE) is a highly multidisciplinary field that focuses on novel regenerative treatments and seeks to tackle problems relating to tissue growth both in vitro and in vivo. These issues currently involve the replacement and regeneration of defective tissues, as well as drug testing and other related bioapplications. The key approach in TE is to employ artificial structures (scaffolds) to support tissue development; these constructs should be capable of hosting, protecting and releasing bioactives that guide cellular behaviour. A straightforward approach to integrating bioactives into the scaffolds is discussed utilising polyelectrolyte multilayer capsules (PEMCs). Herein, this review illustrates the recent progress in the use of CaCO_3_ vaterite-templated PEMCs for the fabrication of functional scaffolds for TE applications, including bone TE as one of the main targets of PEMCs. Approaches for PEMC integration into scaffolds is addressed, taking into account the formulation, advantages, and disadvantages of such PEMCs, together with future perspectives of such architectures.

## 1. Introduction

Defect or loss of tissues are mainly caused either by injuries/traumas, diseases or congenital abnormalities, and are major human health issues that many people may suffer from in daily life. The grafting of a tissue or organ, prosthetic substitutions, implants and other medical devices [1,2,3] are among of the conventional strategies developed in biomedicine in order to face the replacement of malfunctioning tissues, cells or organs. However, these conventional treatments have serious drawbacks, such as donor shortage or limited applicability to restore all the functions of the relevant defective organ, cell or tissue; resulting in little-to-no success of the total recovery of respective tissues or organs [3,4]. Therefore, emerging attention has been shifted to the development of biomaterials that are able to stimulate the regenerative mechanisms in vivo for the maintenance and recovery of defective tissues. This approach is the main strategy in TE, the multidisciplinary field that focuses on the development of tissues and restoration of functions of damaged tissues [5,6,7].

TE is based upon the fabrication of artificial scaffolds, of which has attracted attention from multiple different disciplines across the sciences and clinical settings. Artificial scaffolds are key components in the successful application of TE, they provide a support/anchor for cells to adhere and proliferate upon, in order to aid the regenerative process [8,9]. 3D scaffolds must replicate properties of the extracellular matrix (ECM) and mimic the behaviour of tissues in the body. They must be able to guide the organisation of tissues and modulate the cell proliferation and differentiation [10,11,12]. This is achieved by the formation and loading of scaffolds with bioactives (e.g., growth factors (GFs), cytokines, hormones, etc.) [13,14], via tailoring the scaffolding materials to simulate those present within the ECM, and to replicate its mechanical properties [11,15]. Artificial scaffolds must be multifunctional, biodegradable, biocompatible, hold a developed porous structure, maintain metabolic activity of cells, and possess suitable mechanical properties and surface roughness to allow the hosting of cells and provide structural integrity [2,9,16]. These properties strongly depend on the choice of the scaffolding materials. The materials must be non-toxic, biodegradable and possess the desired mechanical properties [17].

Significant progress has been made for the design of scaffolds for the repair of a variety of tissues using a plethora of materials, including ceramics, both natural and synthetic polymers, and nanocellulose [18,19]. Scaffolds composed of ceramics, such as hydroxyapatite (HA) [20,21] and tri-calcium phosphate (TCP) [22], are used for the regeneration of hard tissues, and are typically very stiff, e.g., bone tissues [23]. Furthermore, very popular FDA-approved synthetic polyesters such as polyglycolic acid (PGA) [24,25], polylactic-co-glycolic acid (PLGA) [26,27] and poly-lactic acid (PLA) [28,29] have been successfully implemented into many scaffolding applications. PLA can exist as either the D-, L-lactide enantiomers, or a mixture of both (PLDA), of which L-lactide is popular in TE applications due to its slow degradation rate [30]. Moreover, further polymers—such as polyether ether ketone (PEEK) [31], polyimide [32], and polyurethanes [33,34]—have been used as successful scaffolding materials for the repair of a variety of tissues. Naturally occurring biomaterials—for instance, collagen [35,36,37], chitosan (CHT) [38,39], alginate [40,41], fibrinogen [42,43], and gelatin [44,45]—have also seen recent work as the main components of scaffolds, proving to enhance cell adhesion due to the recognition features they exhibit. These natural polymer-based scaffolds, however, often hold poor mechanical properties [46,47,48]. Hence, a combination of these materials is preferable to provide support for both soft and hard tissues, with a range of mechanical properties and possible facilitated cellular activities [49,50,51,52].

Many techniques have already been utilised for the construction of scaffolds that exhibit the required properties (porosity, stability, swelling capacity, etc.) for use in TE, including emerging microfluidic-based approaches [53]. Computer-aided design and the increasing popularity of 3D printing are proving themselves as innovative assistive methods in designing scaffolding architectures using a variety of methods [54,55,56]. Namely, solvent/casting particulate leaching [57,58], emulsion freeze drying [59], microsphere sintering [60], electrospinning [61,62], gas foaming [63,64], and thermally induced phase separation [65] are examples of the numerous traditional techniques that have been employed for the fabrication of 3D scaffolds; providing a high porosity, with an interconnected structure that can guide the organisation of neo-tissues in the human body. However, many of these methods, although advantageous in terms of high potential porosities and low-cost production, possess drawbacks in relation to the low control of porosity, long fabrication times, and the harsh conditions that are often used for the preparation of scaffolds: too high or low a temperature, organic/toxic solvents, active chemicals, additives (including surfactants), harmful residues, etc. These harsh conditions will prove damaging to the bioactive in question [66,67].

Indeed, the successful retention and controlled release of bioactives from scaffolds is still a challenge. The simplest approach to incorporate bioactive entities into scaffolds is to incorporate them directly into the scaffolding material [68]. Nevertheless, approaches based on the direct immobilisation of bioactives into scaffolds typically lack protection of bioactives against enzymatic degradation. This direct immobilisation method results in the retention of bioactives via a poor physical interaction with the scaffolding material itself, resulting in the continuous release of the bioactives due to an established equilibrium between free and bound bioactives within the surrounding microenvironment. Altogether, if the bioactive is directly integrated into the scaffolding material, the release dynamics of the bioactive are not guaranteed [68,69]. Moreover, the release of the biomolecules at a specific site at the required time, will not be controlled nor modulated accurately using this approach, as this interaction strongly depends on the properties of the scaffolding material and bioactive of interest (e.g., hydrophobicity, surface roughness, porosity, etc.) [70,71].

New and emerging strategies have been recently developed which seek to prolong and control the release of bioactives from scaffolds and achieve the loading of bioactives in soft conditions. This will lead to the development of a new generation of scaffolds able to satisfy TE requirements and the tailored release of the bioactives of interest. The loading of the bioactives into particle-based carriers is a straightforward approach that has received recent accumulative attention. Particle-based carriers can act as protecting agents to the payload whilst being sensitive to different stimuli (e.g., magnetic field [72], ultrasound [73], light irradiation [74], enzymatic degradation [75], etc.) in order to induce controlled release of the bioactives [76,77]. A number of particulate-based approaches have been recently carried out for bioactive delivery from scaffolds; for instance, from PLGA [78,79], gelatin [80], alginate [81], silica-based [82,83] particles, as well as liposomes [84,85], etc. These carriers can be integrated into the pre-fabricated scaffolds or integrated during the assembly of the scaffolds [86]. Among examples of such a carrier that can improve the respective properties of scaffolds used for TE applications are multilayer polymeric capsules produced using the layer-by-layer (LbL) approach. Templated upon sacrificial core templates, PEMCs may be formed from a variety of core materials, including inorganic (e.g., carbonates, silica), organic (e.g., melamine formaldehyde, polystyrene) and biological (e.g., erythrocyte cells) templates [87,88,89]; CaCO_3_, of which, is highly attractive in terms of its high biocompatibility, low cost production and its ability to host large amounts of cargo under soft conditions [90]; properties ideal for scaffold integration. LbL-assembled polymer capsules (i.e., PEMCs) are delivery vehicles with highly tuneable properties, which can be readily combined with other materials to produce hybrid structures useful in a variety of bio-applications [91,92,93]. PEMCs integrated into scaffolds can serve as protective reservoirs for bioactives, and the bioactive release can be tuned with high precision [94]. Additionally, PEMCs may be tailored to host targeted specific cells via altering the multilayer composition or via cross-linking to change their mechanical properties [95,96,97,98,99].

Microencapsulation into PEMCs has been shown to be one of most promising techniques for the controlled delivery of bioactives [100] due to their many tunable properties and controlled modes of release [101]. Microencapsulation can be defined as the entrapment of molecules of interest into a particle within the particle size range typically from hundreds of nanometers [102,103] to tens of microns [104,105]. The utilised delivery carriers can be readily added into the pre-fabricated scaffold or during the fabrication process. This strategy has attracted much recent interest, as these carriers can aid in the protection of the bioactive of intertest from effects such as proteolysis, denaturation and loss in bioactivity, whilst also prolonging the release [106,107].

Henceforth, this review seeks to provide an overview of the role of vaterite CaCO_3_-templated PEMCs in scaffold-based tissue engineering. This topic is not widely presented within the literature, as up to now there are few studies on this emerging topic. Firstly, the need for bioactive delivery is discussed, followed by the formation of LbL structures and, examples of the applications of PEMCs—taking into account the encapsulation and controlled release dynamics of these structures, as well as their potential hindrances in scaffold integration. Altogether, this review serves to highlight perspectives for the use of the PEMC-based scaffolds for future TE applications, focusing on the typical criteria for such hybrid scaffolds aforementioned.

### The Need for Controlled Delivery of Bioactives

The sustained release of bioactive agents (e.g., GFs and other signalling molecules) in a spatiotemporally regulated manner is one the major functions that a scaffold should possess for successful use in TE. After cell seeding and initiation of tissue growth, the scaffold should degrade as soon as the sufficient regeneration of tissue occurs. The regeneration process begins by stimulating the cell with appropriate GFs (with a controlled dosage).

**Figure 1:**
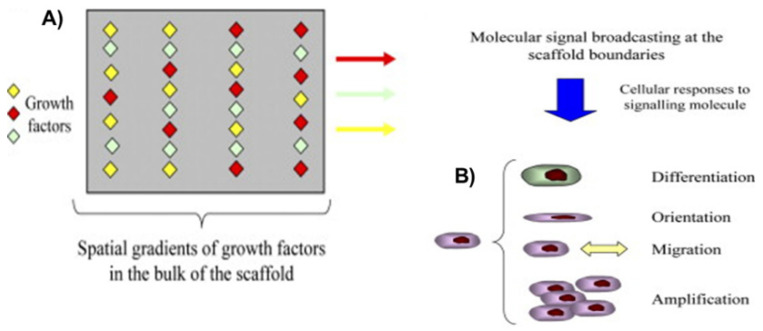
Schematics showing the role of growth factors (GFs) in tissue engineering (TE); (**A**) scaffold incorporated with GFs induces molecular signal and (**B**) molecular signal-induced cellular activities for the healing process of the defective tissue. This schematic is taken from reference [69], copyright© 2008, Elsevier.

GFs are molecules that transmit signals to regulate cell activities (Figure 1), including cellular adhesion, proliferation, migration and differentiation, and hence partake in the growth and repair of a variety of tissues [108]. Examples of the abundance of GFs that have previously been delivered successfully include: basic fibroblast growth factor [109,110], transforming growth factor β3 [111], nerve growth factor [112], vascular endothelial growth factor [113], insulin-like growth factor [114], platelet-derived growth factor [115], and many more [116]. Under physical circumstances, endogenous cells produce GFs, but for the tissue restoration or tissue growth in situ, they must be delivered exogenously [108,117]. The delivery of GFs is one of the central tasks in TE and, simultaneously, remains one of the major challenges in TE. Bioactives that are induced exogenously have the tendency to move rapidly away from the site of regeneration, and also have issues with the maintenance of their bioactivity—leading to the failure of the healing process [106]. Thus, the controlled release of single or multiple GFs at a specific cell or tissue for prolonged periods of time is one of the biggest challenges in TE.

Recently, various approaches have been developed for the delivery of growth factors to achieve sequential and multifactor delivery [49] to mimic signalling taking place in nature, i.e., in the real ECM. The incorporation of bioactives into the polymeric scaffolds directly during the construction of scaffolds is the simplest strategy. However, this might be ineffective, especially since the biological activity and integrity of the growth factors cannot be retained if loaded into the scaffolds using harsh preparation conditions previously mentioned. The next sections focus on one of most attractive delivery carriers, PEMCs, and their employments for the formulation of functional scaffolds to tackle the hurdles of bioactive protection and controlled release.

## 2. The Fabrication of PEMC Structures

**Figure 2:**
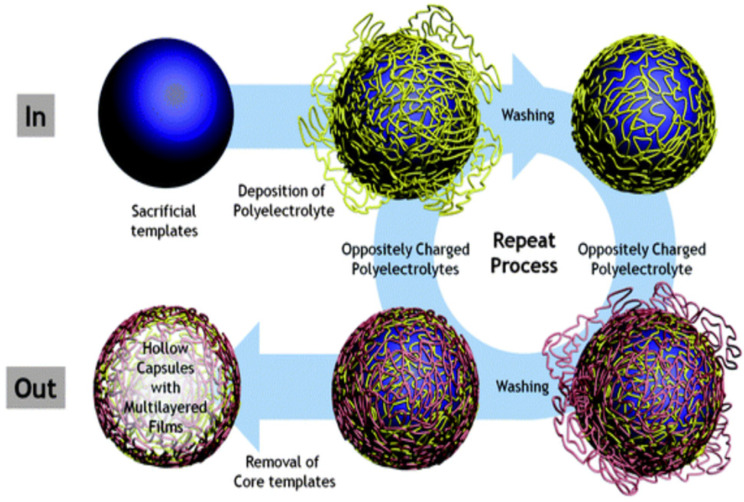
Schematic representation of polyelectrolyte multilayer capsules (PEMCs) formation using the polymer LbL deposition technique. This schematic is taken with permission from reference [119], copyright© 2011, Royal Society of Chemistry.

The LbL approach of polymer assembly was introduced around three decades ago and has proven, up to now, to be a very powerful tool for the fabrication of planar films, and also 3D structures—PEMCs. PEMCs have a high degree of control over their physicochemical properties such as morphology, size, composition, etc. [118]. The LbL method is based on the alternative deposition of oppositely charged polyelectrolytes (i.e., polymers carrying a permanent charge). A positively charged polyelectrolyte is adsorbed onto the negatively charged polyelectrolyte and vice versa, forming a thin film that is often called multilayers [118]. This thin film can be formed not only onto planar surfaces but also onto the surface of a 3D core resulting in the formation of a PEMC, as shown in Figure 2 [119]. The formation of PEMCs is typically achieved through stepwise adsorption of oppositely charged polyelectrolytes onto this sacrificial core followed by elimination of the core at conditions where the multilayers (Figure 2) are stable, and the core is unstable. The core is typically a colloidal particle of potentially any shape. The core may be a well-defined colloidal symmetric particle or a particle with an undefined morphology such as a protein aggregate [120,121].

**Figure 3:**
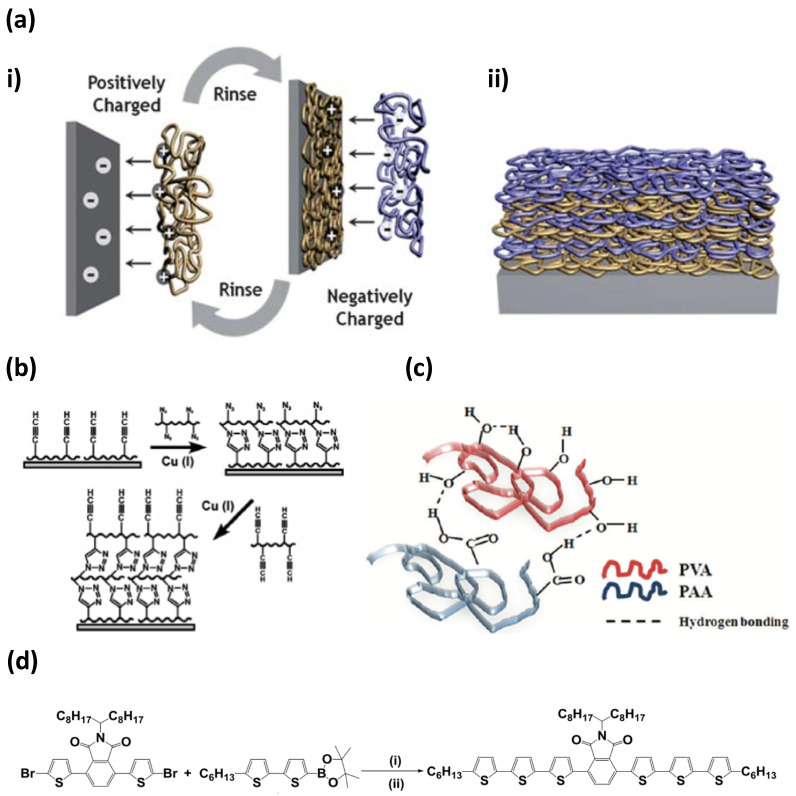
Schematics demonstrating the different forces that can occur during multilayer formation. (**a**) Electrostatic attraction, (**b**) covalent interactions, (**c**) hydrogen bonding, and (**d**) via charge-transfer interactions. (**a**) is taken with permission from reference [119], copyright© 2011, Royal Society of Chemistry. (**b**) is taken with permission from reference [128], copyright© 2006, American Chemical Society. (**c**) is taken with permission from reference [129], copyright© 2012, American Chemical Society. (**d**) is taken with permission from reference [130], copyright© 2017, Elsevier.

There are five main types of LbL deposition: the immersive [122], spin [123], spray [124], electromagnetic [125], and fluidic [126] techniques. In many cases, the main driving force responsible for the formation of multilayers is an electrostatic attraction. However, in general, the overall forces taking place between polymers in PEMs involves a net of electrostatics, hydrogen bonding, covalent bonding (if specific chemical reaction take place), specific recognition, hydrophobic interactions, etc. [121]. Examples of such interactions are illustrated in Figure 3.

The utilisation of the LbL approach offers a considerable control over the uniformity of coating and thickness of the planar or curved PEMs (PEMCs) and also helps to address the aggregating issues (bridging PEMCs together) [118,119,120,121,127].

### 2.1. Multilayer Shell Composition

The choice of the shell components for the successful formation of the multilayers is of crucial importance. Examples of the properties that the multilayer components must exhibit are the ability to produce a layer that enhances the electrostatic forces between it and the core material, to be non-reactive towards the core nor with the potentially loaded bioactives, and to be chemically non-toxic (biocompatible). Thus, this aids in the achievement of the desired scaffold properties such as integral stability, permeability, mechanical properties, etc [100]. Moreover, the chemical nature and molecular weight of the polymers must also be considered [131]. There is a high risk for a low-molecular-weight polymer to diffuse into the interior of the porous vaterite CaCO_3_ core. As a result, a thin multilayer film will not be created on the surface of the core, but rather a thick shell will be formed with a deep interconnected polymer matrix within the core. This has an impact on the formation of the desired PEMCs and affects their properties. On the other hand, heavier polymers with higher molecular weights are less capable to permeate the porous structure and hence they are adsorbed exclusively onto the core surface, leading to the formation of a capsule with no polymer matrix [132]. Therefore, for successful formation of hollow capsules, polymers with high molecular weight are more preferable than polymers with lower molecular weight. Simultaneously, hollow capsules can have better defined but thinner shells with varying mechanical resistances that are crucial to forming scaffolds with high integrity and mechanical stability.

### 2.2. Sacrificial Cores

Initially, PEMCs were formed using organic microparticles as the core material, such as polystyrene (PS) [133] and melamine formaldehyde (MF) [134] latexes. For the elimination of these organic cores, pure organic solvents or rather concentrated acids (0.1 M HCl) are required, which obstructs their use for bioapplications [133,135]. However, inorganic templates have become increasingly popular, including silica nanoparticles (SNPs) [136,137]. SNPs were the first inorganic cores investigated and were predicted to be superior candidates to PS and MF cores. However, due to aggregation problems and acidic elimination conditions (HF), it was suggested that SNPs are not always suitable materials for bioapplications [137]. Carbonate cores are also potential candidates for PEMC formation, including CaCO_3_, MnCO_3_ [133] and CdCO_3_ [138]. CaCO_3_, however, is often seen as the most favourable sacrificial core, having multiple benefits. These include mild dissolution conditions (required for bioapplications), high loading capacities due to its mesoporous structure, biocompatibility, and low-cost production [139]. Despite a number of advantages, the main drawback that CaCO_3_ cores suffer from, is the difficulty to produce monodisperse crystals, as production is very sensitive to many environmental parameters [140,141,142]. The next section will be devoted to the synthesis and properties of the vaterite CaCO_3_ crystals.

#### Vaterite CaCO_3_ Crystals

**Figure 4:**
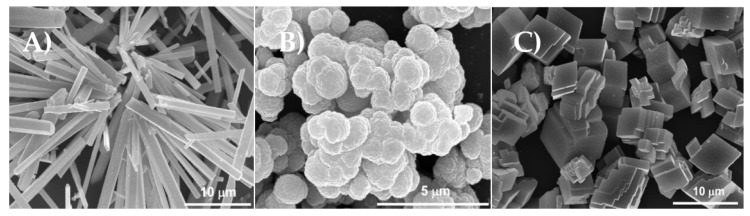
SEM images of three different polymorphs of CaCO_3_ crystals: (**A**) aragonite; (**B**) vaterite and (**C**) calcite crystals. This figure is taken from reference [146], copyright© 2010, American Chemical Society.

CaCO_3_ crystals are inorganic particles and exist as three main polymorphs, i.e., calcite, aragonite and vaterite. The calcite polymorph occurs naturally and is the most stable configuration [139,143,144,145]. The three polymorphs can be easily distinguished based upon their morphology. Aragonite crystals possess a needle-like shape, vaterite crystals are typically spherical crystals, and calcite is cubic-like in shape, as illustrated in Figure 4.

Vaterite CaCO_3_ crystals are the most popular sacrificial cores for the encapsulation of bioactives due to their porous structure, juxtaposed to aragonite and calcite; even though it is, comparatively, the least stable CaCO_3_ polymorph. After more than a decade after the introduction of PEMCs, it can be expected that vaterite CaCO_3_ crystals are of the most suitable sacrificial cores that can be used as templates for the formation of scaffolds, and nowadays, their use is well justified [139,147]. As briefly aforementioned, due to the inherent mesoporous crystal structure and biocompatibility of the core, vaterite possesses a high loading capacity, because of which, bioactive molecules can be readily encapsulated into their interior, at bio-friendly conditions. Consequently, vaterite holds many implications for TE and bioapplications in general. Moreover, the possibility to control the size of vaterite crystals within the range of nano- to micro-metres and adjust their shape [140], as well as porosity [148,149], clearly demonstrates that these cores may be used as versatile microcarriers for advanced drug delivery, and give large scope to control the morphology and internal structure of PEMCs templated upon them. Vaterite is also able to undergo dissolution at mild conditions; it can be readily extracted either by adding ethylenediaminetetraacetic acid (EDTA) or citric acid at neutral pH, both of which are chelating agents for the extraction of Ca^2+^ ions. The cores can also be readily dissolved at pHs below and close-to neutral due to the high solubility of CaCO_3_ in slightly acidic mediums.

**Figure 5:**
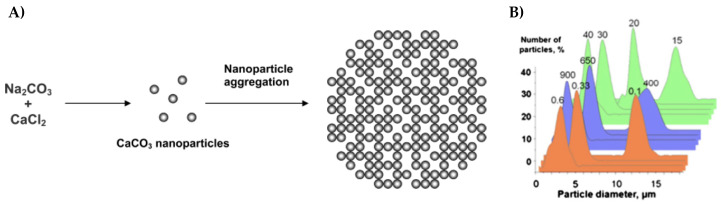
(**A**) Schematic demonstrating the formation of a CaCO_3_ vaterite crystal and (**B**) vaterite CaCO_3_ crystal size distribution as a function of preparation conditions (salt concentration (c), speed (v) and time (t) of salt stirring). Parameter variations are on top of the curves. Orange—variation of salt concentration (v = 650 rpm, t = 30 s); violet—variation of stirring speed (c = 0.33 M, t = 30 s); green—variation of stirring time (c = 0.33 M, v = 650 rpm). (**A**) is taken with permission from reference [160], copyright© 2004, American Chemical Society. (**B**) is taken with permission from reference [139], copyright© 2014, Elsevier. This was adapted from reference [152], copyright© 2012, John Wiley and Sons.

The process for the synthesis of vaterite crystals is rather simple and inexpensive. Vaterite crystals are often produced by the rapid mixing of two supersaturated precursor salts (e.g., sodium carbonate (NaCO_3_) and calcium chloride (CaCl_2_)), as illustrated in Figure 5A. After the appropriate agitation of the salt solutions, the growth of the crystals begins. After growth, the precipitate is finally filtered out resulting in spherical shaped vaterite crystals. However, the difficultly to obtain non-aggregated and monodisperse crystals is still pronounced. Experimental conditions, such as the concentration of the respective precursor salts, the pH, the agitation time and speed, and the temperature, can affect the final crystal size and morphology [150,151,152,153], as illustrated in Figure 5B. For instance, if precursor salt concentrations are increased whilst the remaining parameters remain constant, smaller vaterite crystals will be produced. Moreover, the same result would be expected when the either the agitation time or the agitation speed is increased whilst the remaining conditions are kept constant [139,152]. This is related to the formation of nuclei (nano-sized initial crystallites which aggregate to form the secondary crystal), which is dependent on the salt concentration and agitation conditions, and contribute to the heterogenous growth of CaCO_3_ [154].

However, a limitation for vaterite use as sacrificial cores for PEMC formation that has been reported, is the vaterite-to-calcite re-crystallisation process [155]. This occurs when vaterite is exposed to aqueous solutions for an extended period of time, usually hours to tens of hours [139,156]. The re-crystallisation may be a problem if long-term stability is required. However, the re-crystallisation may be advantageous if desired; it is one of the mechanisms of payload release from the vaterite cores, since the non-porous calcite it re-crystallises to has a much lower surface area compared to the mesoporous vaterite [157]. Moreover, the re-crystallisation rate can be adjusted via the modification of vaterite crystals with polymers [158,159].

## 3. Applications of PEMCs as Delivery Carriers

**Figure 6:**
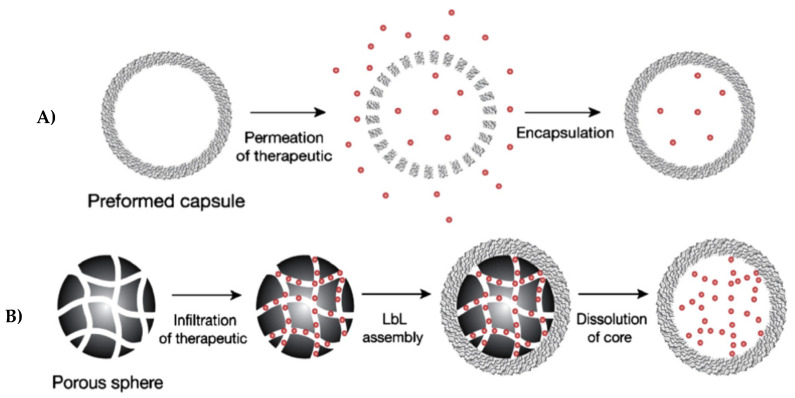
Schematics for the encapsulation of molecules of interest (e.g., bioactives) into hollow PEMCs: (**A**) loading into pre-formed PEMC and (**B**) incorporation into a porous sacrificial core utilized to assemble the PEMC. This figure is taken with permission from reference [163], copyright© 2006, Elsevier.

The carriers that have been found to be able to satisfy the requirements of both drug delivery and TE are PEMCs of two types—hollow- and matrix-type capsules. Hollow capsules have a well-defined multilayer shell that serves as a barrier to keep the cargo inside the capsule and release it once the barrier is no-longer intact (e.g., from the formation of a pore or pores in the shell). Matrix-type capsules are made fully of multilayers, and the bioactive is bound to the multilayers typically via interaction with free charged groups of polyelectrolytes [161,162]. The formation of a hollow or matrix-type capsule can easy be tuned by the porosity of vaterite CaCO_3_ crystals used to form the capsules [149]. For more porous crystals, the polyelectrolytes can permeate deeper into the pores, and therefore form a polymeric matrix. Figure 6 illustrates two diagrams describing two main approaches for the entrapment of bioactives into the lumen of hollow capsules. The approaches are based on: (i) the loading into pre-formed capsules via change in the capsule shell permeability and (ii) incorporation into a porous material served as sacrificial core to assemble a hollow capsule (i.e., post- and pre-loading, respectively) [163].

PEMCs are excellent candidates for: (i) the encapsulation of bioactives/therapeutics for the protection of the therapeutics either from external or internal stimuli; (ii) specific cellular interaction/internalization by tailoring the properties and surface chemistry of the capsules; and (iii) sustained release of the payload after application of a specific stimulus [163] that changes the PEMC’s physicochemical properties when reaching their target site-attributes of which are the reason for their surge in popularity in recent years; these are the criteria a PEMC should aim to meet for use as a drug delivery vehicle.

In further detail, the release of the bioactives can be adjusted via changing certain parameters that the multilayer shell is sensitive to. The majority of multilayers are shown to be responsive to both preparation conditions and environmental changes post-preparation (e.g., temperature [164], ionic strength, pH, etc). The charge density of each polyelectrolyte that is used to form the shell membrane can be altered by varying the pH and ionic strength during PEMC formation or after fabrication [165,166,167,168]. This results in a change in the charge balance within the multilayers, and thus affects the balance of attractive and repulsive forces between polymers, leading to rearrangement of the polymer chain configurations [167]. This, consequently, changes the permeability of the multilayer shell (more or fewer voids/pores in multilayers). For instance, the capsule shell is in “open” state with higher permeability of the shell and the biomolecules can diffuse inside the capsules or, if there already inside, to be released. When the pH is changed, the capsule shell can switch to the state with a lower permeability (i.e., “closed” state) when the biomolecules will be entrapped inside or the release will be stopped, respectively [169]. However, overdosing of these stimuli (e.g., pH, ionic strength, temperature, etc.) can result in the disassembly of the multilayers [170,171].

**Figure 7:**
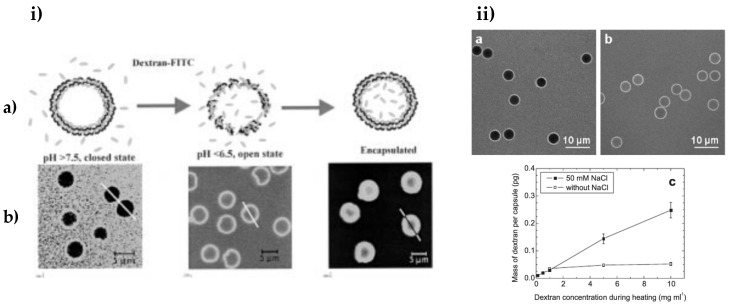
The permeability of PEMCs varying with the (**i**) pH and (**ii**) ionic strength. (**i**) The loading of FITC-dextran inside PSS/PAH PEMCs and release via decreasing the pH, where (a) illustrates a schematic diagram of the closed state of the capsules at higher pH value and the open state at lower pH value; with (b) their corresponding confocal laser scanning microscopy (CLSM) images of PEMCs. (**ii**) CLSM images of incubation of poly (diallyldimethylammonium chloride) (PDADMAC)/PSS capsules without adding any salt (a) and in the presence of salt (b,c) Graph showing the amount of incorporated FITC-dextran inside capsules with and without salt. (**i**) is taken with permission from reference [172], copyright© 2001, John Wiley and Sons. (**ii**) is taken with permission from reference [173], copyright© 2007, John Wiley and Sons.

For instance, a study [172] demonstrates that, at rather high pH values (pH above 7.5), the hollow poly(allylamine hydrochloride) (PAH)/poly(styrene sulfonate) (PSS) capsules prepared are impermeable (“closed” state) and at lower pH (below 6.5) the shells are permeable (“open” state). This is due to an increase in the protonation of PAH primary amino groups, and hence there are more repulsive interactions between the like positive charges of those groups, and thus more voids/pores within the multilayers. If the pH is shifted back to higher values (pH more than 7.5), the dextran-labelled with fluorescein isothiocyanate (FITC) that has permeated within the capsules will be entrapped as soon as the capsule shell will change its permeability. The permeability of shell membrane by varying the pH and dextran-FITC encapsulation are demonstrated in Figure 7i.

Köhler and Sukhorukov [173] reported that a change in ionic strength can result in significant change of the capsule permeability. At low concentrations of salt (NaCl), the diffusion of the FITC-dextran was prevented as the shell membrane was closed (Figure 7(iia)), when, at an increased ionic strength of 50 mM after extra salt was added to the system, the capsules became permeable (Figure 7(iib)). At a higher ionic strength, an adequate amount of the labelled dextran was incorporated inside the capsules in comparison with that at a low salt concentration, illustrated in Figure 7(iic). This is due to the increased screening of permanent charges along the polyelectrolyte backbone by counter ions arising from salt. This results in drastically reduced intrachain repulsion, resulting in reduced electrostatic attraction between the respective polymers, and may lead to polymer coiling. This, in turn leads to a more porous (i.e., permeable) multilayer structure. Hence, the multilayers consisting of polyelectrolytes (both bio and synthetic) are very attractive in regard to their permeability and other respective properties, which can be easily tailored by the variation of a number of deposited layers [174], as well as deposition conditions such as pH, ionic strength and temperature (reviews [175,176] and references therein).

Furthermore, PEMCs are ideal vehicles for binding selectively at a specific targeted cell. This is due to the easily tunable surface chemistry of the multilayer shells of PEMCs. The shell can be modified for cell specific interaction by functionalising multilayers with peptides, proteins, nucleic acids (e.g., DNA, RNA, etc.) [177] and metal nanoparticles (NPs) (e.g., gold NPs) [178,179] as the ligands themselves or additives that are bound to specific ligands. These ligands can interact at specific sites (receptors) at the targeted cell and potentially lead to the internalisation of the capsules inside the targeted cell, that is typically driven by endocytosis. The intracellular delivery of PEMCs is out of the scope of this review, but shows interesting applications of PEMCs.

**Figure 8:**
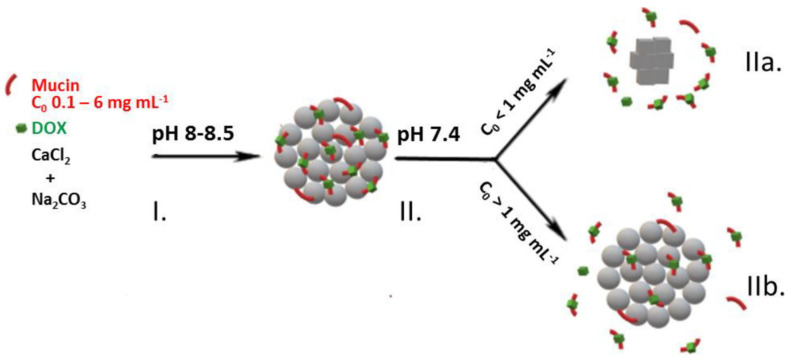
Schematic of the co-synthesis of CaCO_3_-mucin hybrid crystals containing doxorubicin (**I**) and the release of doxorubicin at physiologically relevant conditions (**II**) via diffusion of mucin-doxorubicin complex out due to the re-crystallisation of the CaCO_3_ crystals to calcite (**IIa**) and the complex diffusion out of the pores of the vaterite crystals (**IIb**). This schematic is taken with permission from reference [183], copyright© 2019, Elsevier.

For any kind of PEMC-based delivery (intra- or extra-cellular), the crucial step is to load bioactives inside capsules, and the approaches described above can be used to load and release bioactives with rather high molecular weights, typically more than 5–10 kDa. This is limited to the size of voids/pores in the capsule shell, as those smaller molecules, such as low-molecular-weight drugs, (up to a few kDa) can permeate through the capsule shell [180]. Therefore, PEMCs are ideal carriers only for the loading of biomacromolecules if the shell permeability is used as a factor to control release rate. Another approach has been introduced to load small drugs (even below 1 kDa) via the pre-loading of vaterite CaCO_3_ crystals with a high-molecular-weight molecule that has a good affinity to the small drug in question. These hybrid crystals can be used to form PEMCs with desired properties [181]. An example includes the loading of doxorubicin into vaterite crystals pre-loaded with mucin (Figure 8). Mucin loading was performed via co-synthesis [90,182]; i.e., when mucin was added during vaterite crystal growth and entrapped within the crystals in a very high amount [183]. This ensures that a rather large quantity of doxorubicin can be loaded into such composite capsules due to the high electrostatic affinity of doxorubicin to mucin. Significant loading is a prerequisite for prolonged delivery to provide the required sustained doses of the drug. Mucin, of a number of other biologically relevant molecules, can be loaded into the vaterite crystals by co-synthesis or by physical adsorption by allowing the crystals to interact with the biomolecules for sufficient time [184]. Nevertheless, co-synthesis is one of the most promising methods to encapsulate bioactives into vaterite crystals due to a fine control over encapsulation capacity and a high retention of bioactivity [185,186].

The multilayer shell itself can host an incredibly high amount of small charged molecules, including charged drugs. This is mainly driven by the binding of the drug molecule to free (uncompensated by permanent charges of oppositely charged polymer) charged groups of polymers in multilayers [187]. Based on this, the multilayer shell can be stained using fluorescent probes, which are typically small charged molecules [101] but can also host large proteins with molecular weight of a few hundreds of kDa [188]—providing large scope for scaffold-based applications.

The release of bioactives from PEMCs is crucial to achieve the required loading of bioactive within the scaffold to be adjusted with a high resolution in both space and time. For this, the PEMCs may be designed to be able to release a payload remotely. The PEMCs modified by metal NPs, such as AuNPs, can release bioactives through external stimuli such as light [189,190]. Metal NPs deposited onto the surface of capsules can adsorb the light energy and convert it to heat. This can cause a local overheating and cause the loaded material to be released from the PEMC to the targeted cell, with the potential to go to the level of a single cell [191]. PEMCs loaded with proteins, nucleic acids and metal NPs have shown to be effective in cancer treatment and gene therapy [76,176]. Moreover, the NPs can alter the re-crystallisation kinetics of the vaterite-to-calcite transition, and therefore be used to adjust the release kinetics of molecules loaded into the crystals. This is demonstrated with magnetite NPs, of which the authors suggest the presence of magnetite reduced the order of the crystal lattice, accelerating the vaterite dissolution into ions, and hence releasing the encapsulated cargo [157]. Therefore, PEMCs with the ability to host and protect bioactives, as well as to release them on demand, present themselves as very promising candidates for many clinical-oriented medical fields including cancer treatment, combating infection and inflammation, and advanced TE [163,172,176,192,193]. These properties of PEMCs have hence attracted the interest of many, especially in the field of TE [194,195,196], thus, PEMCs hold great implications for use in regenerative processes in vitro and in vivo.

## 4. Problems Associated with PEMCs

The main function and expectation from the PEMCs are that the capsules should be able to host and protect, as well as release bioactives when required. The main mechanism of the release occurring in living systems is based on biodegradation [197,198]. If the PEMCs are made of the appropriate biopolymers, able to degrade into small components in the presence of enzymes, the PEMCs will possess a controlled/programmed release of bioactives that can be enhanced or suppressed depending on the concentration of the enzymes present [199]. However, the main issue is to protect the bioactive components within the PEMCs during transport to the target site, where release should take place. PEMCs often release bioactives due to defects in the multilayer shell. A single defect offering a pore with dimensions larger than that of the bioactive, would obviously result in the inevitable release of the bioactive. Such pores or defects are typically formed during the formation of the PEMCs and can be caused by osmotic shock induced by the dissolution of the sacrificial core [133]. In regard to this, the vaterite CaCO_3_ crystals present themselves as ideal cores for avoidance of this hurdle, as both free and chelated ions released upon the core dissolution will rapidly diffuse through the multilayers with no significant diffusion limitations. As such, the osmotic pressure built up upon dissolution of a CaCO_3_ core is little, but, dependent upon the strength and stability of the multilayers, this may still affect the PEMC integrity. The sections below will focus upon the factors affecting the retention of molecules encapsulated into PEMCs, greatly considering osmotic pressure.

### 4.1. Factors Influencing Bioactive Retention of PEMCs

**Figure 9:**
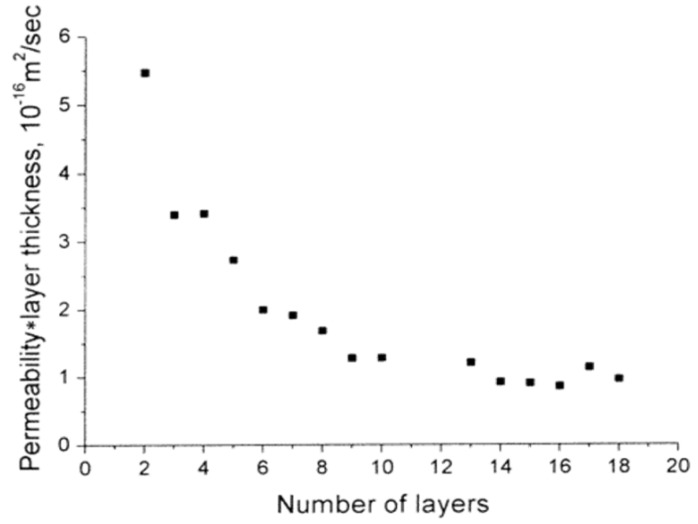
A graph showing the relationship between permeability and number of deposited layers, for (PSS/PAH) PEMCs, when fluorescein is encapsulated within. Permeability determined via the flux of fluorescein through the capsule wall. This figure is taken with permission from reference [203], copyright© 2001, American Chemical Society.

This chapter brings the question: “what factors can affect the efficiency of encapsulation?” to the forefront, in regard to the retention of biomolecules. Critical to this section is the idea that PEMC walls are semi-permeable membranes. This means that the size of the contents (molecular weight, kDa) plays a large role in how well they are encapsulated and retained. The solid but semi-permeable nature of the PEMC wall allows smaller molecules to diffuse outwards with greater ease, compared to that of larger molecules [200]. Thus, many PEMC systems have difficulty retaining smaller biomolecules. To overcome this, the multilayer pore size is controlled [201,202]. The smaller the pore size, the better the PEMC is at retaining smaller biomolecules. It has been proven that greater number of layers in general reduces permeability [203] as demonstrated in Figure 9. This is attributed to the thickness increase with each deposited layer inhibiting the diffusion of the cargo out of the PEMC.

As aforementioned, the ionic strength and pH of the solution making up the local microenvironment of the PEMC also plays a factor in multilayer pore size and layer thickness. Both of these factors can be easily changed during the production of PEMCs. An emerging method of controlling pore size, heat treatment, has been shown to reduce capsule diameter, increase capsule wall thickness and significantly reduce pore size [204,205]. This occurs via the annealing and resealing of the multilayers. This process means that the pore permeability can be said to be dependent upon the temperature. The temperature of annealing is dependent upon the thermal energy required for the polymers to separate, i.e., break their electrostatic bonds [206]. At a temperature above the temperature of phase transition of the multilayers (from solid to liquid phases), the dynamics of multilayers are significantly enhanced, and polymers migrate from one binding site to another. This offers the option for the multilayer structure to re-organise into a more ideal state—all permanent charges on polymer backbones are fully compensated by oppositely charged polymers (intrinsic charge compensation). This reduces the number of pores, and therefore reduces the multilayer permeability (temperature induced annealing).

Modifying the polymer structure also has an effect on the pore size. As discussed above, physical cross linking (intrinsic charge compensation) has an influence on pore size; this can be influenced by the orientation and the number of crosslinking groups of the polymers being used. Moreover, hydrophobicity is influential on the multilayer permeability [207], it is reported that the esterification of the polymers used increased the hydrophobic nature of the multilayer structure and thus decreased permeability [208]. This suggests that different polymer combinations and deposition conditions will inherently affect the multilayers’ ability of retaining bioactives. Even if the pore size is of the correct proportion to retain the bioactives, the PEMC shell may still rupture before reaching the target site, and quite often does. This is typically due to physical forces that occur during formation. Discussion of these forces and their importance in the efficiency of retention will be given in the next section.

### 4.2. Role of Osmotic Pressure in PEMC Formation

**Figure 10:**
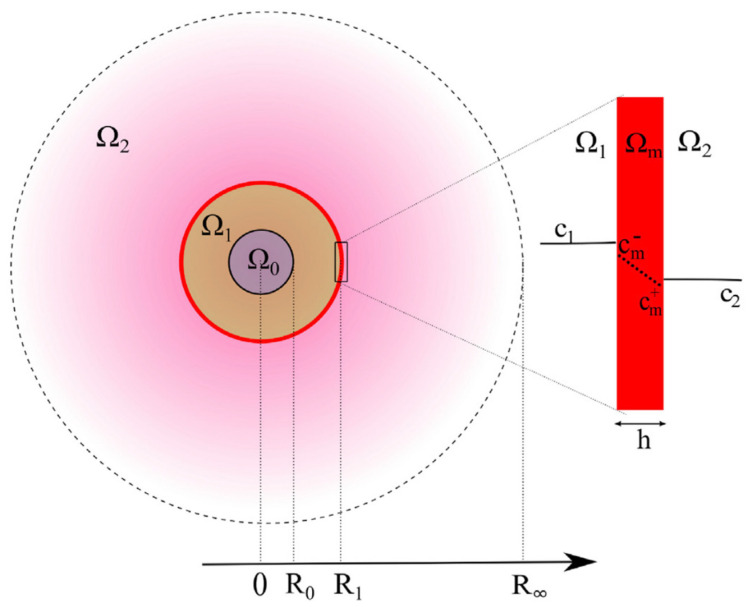
A model of a capsule with several parameters considered during release. 0—is a centre of the particle, R_0_—is distance from the centre to surface of CaCO_3_, R_1_—distance to capsule wall, R_∞_—distance to the edge of diffused contents. Ω_0_—defines the area of undissolved CaCO_3_ loaded with biomolecules, Ω_1_—defines the area where biomolecules are free to move inside the capsule, Ω_2_—represents the system outside of capsules. The red line represents capsule wall, h—is the capsule wall thickness, c_1_ and c_2_—represent the route of travel for flux of contents in and out of capsule, c_m_—represents the route of travel through pores in capsule wall, which is almost never equal to h. This figure is taken with permission from reference [210], copyright© 2018, Elsevier.

A crucial factor to take into consideration for bioactive retention in PEMCs is the principal of osmotic pressure. In Figure 10, an introductory diagram defining the regions of interest during formation and the route of travel a bioactive will take to diffuse out of the capsule is shown. Osmotic pressure will have a great influence on the final capsule diameter and the overall retention; in some systems, the osmotic pressure may prove too much, and the system will burst [209]. The polyelectrolyte walls are very thin in relation to the overall capsule size. In order to successfully encapsulate a bioactive, a PEMC is required to host more bioactive molecules inside their walls than is present outside in the bulk. Therefore, an extreme concentration gradient is created.

This concept is fairly common in cells and the same theory applies. The van ’t Hoff equation (Equation (1)) is used to determine the osmotic pressure [211] on a system.

(1) $$\pi =RT\Sigma \varphi_{s}C_{s}$$

where *π* is osmotic pressure, *R* is the universal gas constant, *T* is absolute temperature, *C_s_* is the molar concentration of solute inside the capsule, and *φ_s_* is the osmotic coefficient, a factor applied to the equation when in non-ideal conditions—for instance, if there is salt in solution or if there is deviation in temperature. From this equation we can draw several conclusions. The flow of water will occur across the membrane from an area of low concentration to an area of high concentration of solute, provided pressures on both sides of the membrane are equal. There is reduced pressure outside of the capsules (Ω_2_ in Figure 10) and an increased pressure inside the capsule (Ω_1_ in Figure 10), as the flow is inwards. This pressure builds up and gradually halts flow into the capsule. Finally, the standard van ’t Hoff equation can only be used when all the bioactive molecules are retained, and the capsule membrane is impermeable to solute. When the membrane is not impermeable, but instead inhibits flow of solute enough to retain the solute for some time (membrane leakage), we see a new coefficient introduced, that is the reflection coefficient, *σ*, as per Equation (2).

(2) $$\sigma =\frac{\pi_{eff}}{RT\varphi_{s}C_{s}}$$

The reflection coefficient can range from a value of 0 to 1. At 0, there is no difference in concentration and there is no flow. At 1, the van ’t Hoff theory applies. When it is somewhere between the two values, C_s_ is no longer static over time. In addition to this, C_s_ may not be equal, which affects the porosity. Hence, a membrane may be semipermeable to solute (e.g., σ = 0.6), as the smaller of the solutes flows outwards and freely enters the bulk solution, and the membrane gradually becomes more impermeable, as only the larger solutes remain encapsulated, and the smaller solutes become very dilute (σ = 0.99). In terms of the kinetic profiles, for a fluorescence profile, this would appear as an exponential decay until a plateau is reached. For capsule diameter over time, a non-linear line is observed, which is correlated to an increase in osmotic pressure until the rupture of the capsule wall takes place.

During capsule formation, there is a process of dissolution of the core, which creates a reduced pressure inside the system. This stimulates an even stronger osmotic flow into the PEMC. So, when modelling osmotic pressure over time, one must consider many different forces at once. For instance, for vaterite CaCO_3_ cores, a reduction in the internal pressure occurs as CaCO_3_ dissolves and unsuccessfully retained solute leaves the system—this also induces osmotic flow. An increase in the internal pressure then occurs as a result of the concentration gradient across the membrane, creating osmotic pressure. Naturally, if the pressure inside a PEMC continues to increase, it will reach a breaking point. At the point at which osmotic pressure in a PEMC becomes so great, it causes rupturing of the PEMC wall, and this is termed the critical osmotic pressure (COP). One study reports the use of vapour pressure osmometry in order to induce osmotic flow into the PEMC to determine what PEMC properties can effect COP [212]. It finds that the upper limit to pressure that a PEMC will withstand can be modelled using the Young’s Modulus, which is a property of materials relating to their elasticity. The three factors that affect the elasticity of the PEMCs are the number of deposited layers, the specific polymers used, and the size of the respective PEMC.

Once a bioactive molecule is released, the control of its rate of release is essential. If the concentration of a molecule is low, its diffusion can be modelled using a Brownian motion. This rate of travel throughout an area is fairly slow, not considering powered biological transport and diffusion phenomena. After release, provided the membrane is ruptured sufficiently, the bioactive is released in a very high concentration in a localised point. Under these circumstances, biomaterials spread at a rate up to 800 times higher than that of the Brownian motion [213]. This can be described by Fick’s second Law of Diffusion (Equation (3)) [210,214].

(3) $$\frac{\partial c_{0}}{\partial t}=D_{0}\nabla^{2}c_{0}$$

where *c*_0_ is the concentration field, *t* is time and *D*_0_ is the diffusion coefficient of the molecule. Due to the rapid diffusion, the PEMC “bursts” and disperses rapidly throughout the area. This is very useful for rapidly delivering bioactives within a specific area, when the PEMC has to release its cargo at once. Some capsules are very elastic, it is reported that when pushed through a microchannel, PSS-loaded microcapsules can recover from extreme deformations [215]. Therefore, if a capsule membrane survives the initial extreme pressure changes during the capsule formation, the osmotic pressure becomes a less viable mechanism for release. The most common mechanisms for incorporating capsules into scaffolds are discussed in the next chapter. For further reading about modelling of the release from PEMCs, see references [210,214].

## 5. PEMC-Based Scaffolds

### 5.1. Scaffolds with Integrated PEMCs

**Figure 11:**
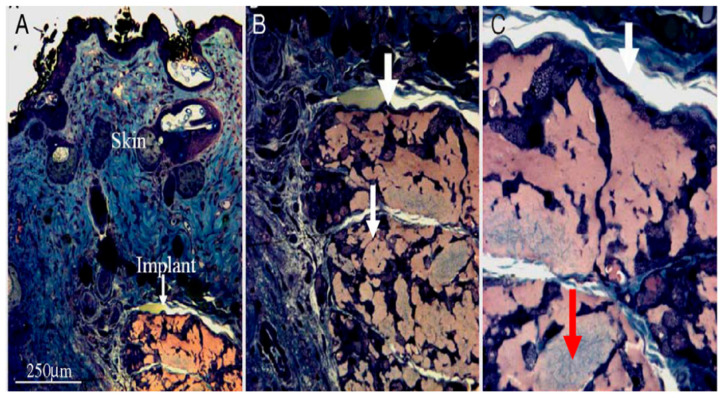
Optical microscopy images taken after in vivo implementation of differentiated EB in the presence of activate capsules (capsules incorporated with GFs) and alginate gel matrix. (**A**) subcutaneous section visualisation of skin (blue) and the implant (orange); (**B**,**C**) enlarged images of implant section representing the vascularisation (white areas) and suggested bone induction (grey area, red arrow). This figure is taken with permission from reference [216].

PEMCs have seen recent use for the formation of a new generation of scaffolds in advanced TE. Recent studies of PEMCs integrated into scaffolds will be discussed in this section. Facca et al. [216] incorporated two GFs, bone morphogenic protein 2 (BMP_2_) and transforming growth factor β1 (TGFβ_1_), into PEMCs composed of poly-L-lysine (PLL) and poly-L-glutamic (PGA) to examine if bone formation in vitro and in vivo could be achieved. A successful induction of bone formation from stem cells was demonstrated in vitro, mediated via these active PEMCs. For the achievement of bone formation in vivo, it was assumed that the embryo bodies (EBs) could grow only in the presence of a proper 3D environment. Therefore, a mixture of active capsules, 3D alginate gel matrix and EB was prepared. After subcutaneous implementation in mice, osteogenic differentiation of EBs was observed, leading to the successful bone formation without the need for a cartilage template. The evidence of bone formation is demonstrated in Figure 11. It is assumed that PEMCs integrated into the suitable 3D artificial matrices can potentially be useful in the process of TE.

Besides, it was observed that scaffolds integrated with GF-loaded PEMCs have the tendency to adopt the properties of the PEMCs. De Cock et al. [94] developed TGFβ_1_-incorporated hollow (heparin (HEP)/poly-L-arginine (PARG))_2_ microcapsules into gelatin cryogel-based scaffolds and reported that the morphology and mechanical properties of the cryogel, as well as the bioactivity of the released GF remained unaffected. Furthermore, del Mercato et al. [217], presented a comparison between the pristine collagen scaffolds and those integrated with vaterite CaCO_3_-templated PARG/dextran sulphate (DS) bio-capsules. It was reported that the structure, porosity, and other physical features (i.e., swelling capacity, stability and mechanical properties) of the PEMC-based scaffolds were similar to those of pristine scaffolds. Therefore, since the scaffolds composed of PEMCs retained the important properties of an ideal/desired scaffold for TE applications, it is suggested that the generation of new scaffolds with controlled bioactive delivery and protection has been achieved via capsule integration in 3D matrices. The vaterite CaCO_3_ cores may also be used as multifunctional particles for the formation of alginate-based scaffolds [218,219]. In this concept, the cores are dispersed into alginate, the cores themselves provide calcium ions that crosslink the alginate, forming a gel. After full dissolution of the cores, an empty pore replaces that of a core, and the size of those pores and their interconnectivity can be controlled using the cores with defined dimensions. Moreover, the cores serve as tools for encapsulation. If a molecule of interest (e.g., GFs) was pre-loaded into the core, the formed pore will be loaded with those molecules (if they do not escape due to rather porous structure of the alginate gel). In general, if the vaterite core is pre-coated with multilayer shell, it will be left after the core removal, giving PEMC-integrated alginate matrices as scaffolds.

### 5.2. Scaffolds Consisting Purely of PEMCs

Recent studies have proven that PEMCs can be used for the formation of scaffolds without the need for their integration into another artificial matrix, such as a polymer gel or polymer bed, required to host cells and develop a tissue. PEMCs can be those units that maintain the integrity of the whole scaffold, especially if the whole scaffold consists only of PEMCs. Henceforth, this section is dedicated to current reports that have developed such scaffolds.

**Figure 12:**
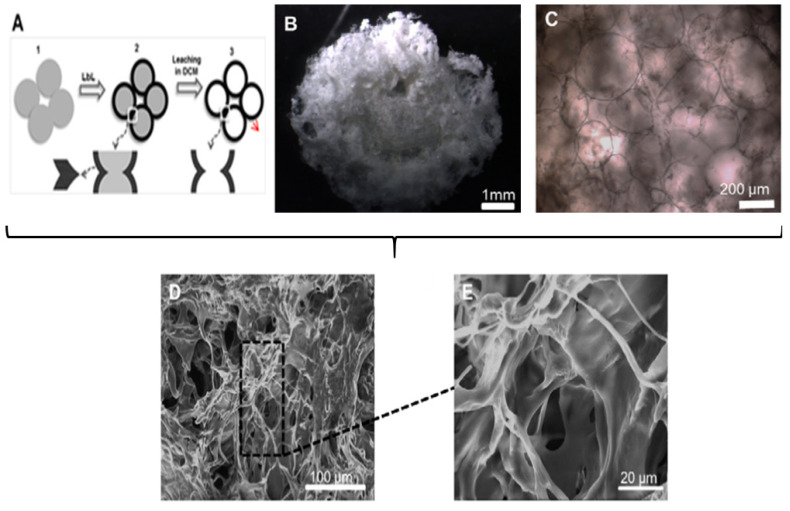
Scaffold characterisation. (**A**) schematic representation of the steps for the production of scaffolds: coating of the paraffin spheres by the LbL approach (CHT/CS multilayers) and leaching of the spheres/core. (**B**) digital photograph of the scaffold. (**C**) optical microscopy image of the scaffold, taken after the leaching of the core. (**D**,**E**) SEM images of a cross-section of the freeze-dried scaffold at two different magnifications. This figure is adapted with permission from reference [220], copyright© 2013, Silva et al.

Silva et al. [220] designed a 3D nano-structured construct based on the biopolymers CHT and chondroitin sulphate (CS) multilayers for cartilage TE. For the fabrication of this advanced scaffold, two steps were used: (i) CHT and CS were templated on packed paraffin spheres via the LbL deposition and, (ii) the spheres were leached out as shown in Figure 12A,B using an organic solvent. In addition, it was reported that the scaffolds had a bubble-like morphology and they adopted the geometry and pore sizes of the paraffin sphere templates, also illustrated in Figure 12B,C. The diameter of the paraffin spheres used as porogens was of 200 µm, which allows the cellular activities to occur with no limitations (i.e., formation of the ECM and cell infiltration). Moreover, after freeze-drying the scaffolds, a porous and interconnected structure was observed in the scanning electron microscope (SEM) images in Figure 12D,E.

**Figure 13:**
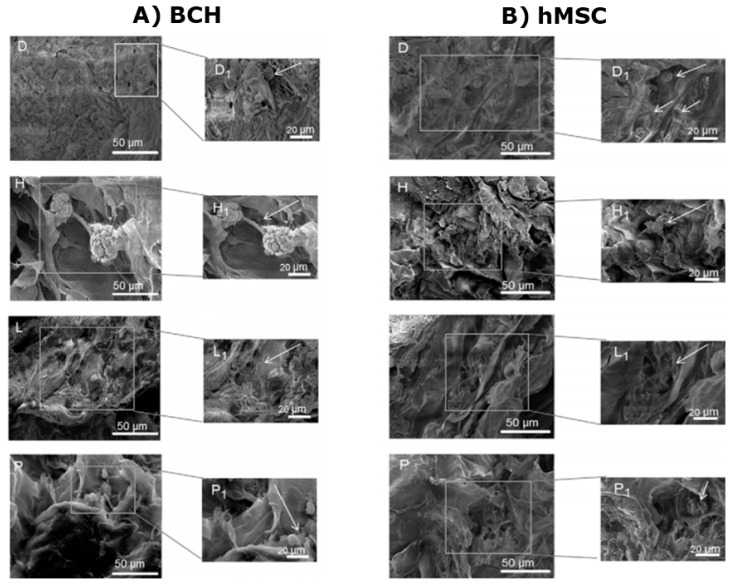
SEM images of the cell seeding on CHT/CS scaffolds after 1-day (D), 3 days (H), 14 days (L) and 21 days (P). (**A**) BCH cells seeded on scaffolds and (**B**) hMSC cells seeded on scaffolds. This figure is taken with permission from reference [220], copyright© 2013, Silva et al.

To illustrate that the scaffolds formed are able to allow the cellular activities such as cellular adhesion and proliferation, two cell-lines were seeded on the prepared scaffolds, i.e., human mesenchymal stem cells (hMSC) and bovine chondrocytes (BCH). Both cells were successfully adhered and proliferated in the CHT/CS scaffolds, as shown in Figure 13. With an increasing number of weeks, the cells were more spread throughout the scaffold, and sulphated glycosaminoglycans were secreted, indicating cartilage formation.

**Figure 14:**
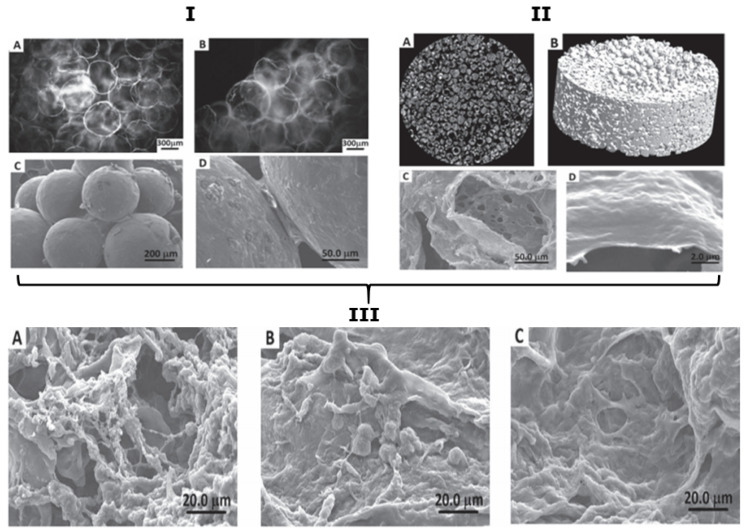
Images illustrating: (**I**) the interconnected PEMCs in 1D (A and B) and in 3D. (**II**) scaffold made of the PEMCs in 2D and 3D (A and B, respectively, from X-ray microtomography) and the internal structures of the scaffold (C and D). (**III**) SEM images of scaffolds with cells for cell culture after 24 h (A), 3 days (B), and 7 days (C). This figure is taken with permission from reference [221], copyright© 2010, John Wiley and Sons.

Sher et al. [221], demonstrated the formation of nano-structured 3D constructs using the same approach as in the previous study. However, in this study, paraffin wax particles were coated with polyanionic alginate and polycationic CHT using the LbL approach, and for the elimination of the cores, the leaching approach was used. The result of this simple multilayer technique was the transformation of a random template assembly into a 3D architecture with interconnected porous multilayer nano-membranes located in a 3D space. The results can be clearly seen in Figure 14I,II. Furthermore, SEM imaging was performed after 24 h, 3 and 7 days of cell culture for the biological evaluation of PEM-based scaffolds with an osteoblast-like cell line. From the SEM images, as illustrated in Figure 14III, it can be observed that in day 1, the cellular adhesion was pronounced (Figure 14(IIIA)). At the 3rd day of cell culture (Figure 14(IIIB)), it can be clearly seen that the spreading of cells has begun, suggesting the scaffold formed provides a favourable environment for cell attachment and proliferation. Finally, after 7 days (Figure 14(IIIC)), the cells were spread to the entire surface of the scaffold. Furthermore, in this study, it was assumed that active GF-loaded PEMCs would be even more favourable in TE, as GFs would facilitate the regenerative process.

### 5.3. Microfluidic Assisted Formation of PEMC-Based Scaffolds

A method that has been used for the formation of 3D scaffolds based on PEMCs is microfluidics. A fluidic assembly can be used for the formation of a thin film via coating the substrates inside a microchannel [126,222]. In terms of the microfluidic assembly, the procedure of the multilayer deposition occurs using fluidic equipment such as fluidic micro-channel for the coating of the substrate, tubes or capillaries for the induction/pull of polymer and washing solutions into microchannel, and a pump (or vacuum) that induces pressure to the system for the sequential movement of polymer and washing solutions through the microchannel.

The idea of fluidic assembly is the formation of coated substrate or more complex 3D structures by pumping solutions into the system to reach the desired material. Moreover, the flow rate of the fluids can be controlled with a high precision. This is indispensable for reproducibility and the enhancement of polymer diffusion, and therefore multilayer formation. Thus, the set-up of this assembly is rather simple, and automated multilayer deposition may be achieved. Furthermore, if everything is at the micro-scale (microfluidic) then very little volume is required, which leads to low-cost procedures and less generated waste (unused/non-adsorbed polymer molecules which may prove difficult to separate from other polymers due to presence of both polymer adsorption and desorption upon multilayer formation). Moreover, by means of fully automatic microfluidics, the assembly of hundreds of layers inside the microchannel can be achieved in a few minutes, or comparable time periods [125,223].

**Figure 15:**
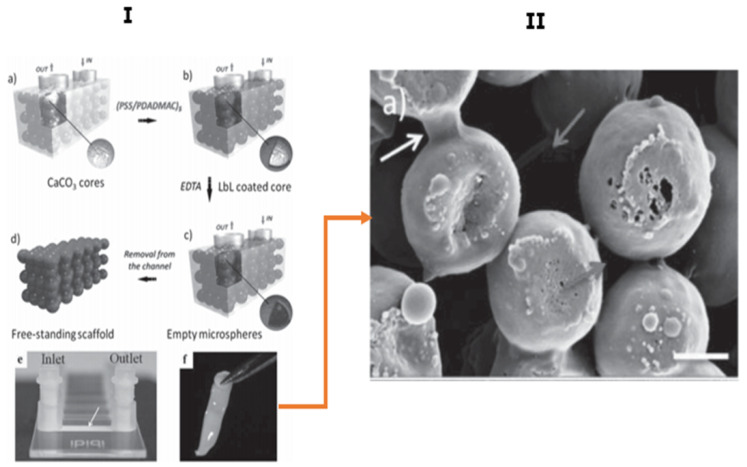
PEMC-based porous scaffold prepared using microfluidics. (**I**) schematic for the procedure of scaffold formation via microfluidic self-assembly deposition and (**II**) SEM image of the packed structure of scaffold; white arrow indicated the junctions between the microspheres and grey arrows, the voids between the spheres due to non-compact (upper arrow) and compact (lower arrow) of core packing. This figure is taken with permission from reference [53], copyright© 2014, John Wiley and Sons.

The pioneering work in the microfluidic assisted formation of the PEMC-based scaffold has been carried out using PDADMAC and PSS polymers, templated upon vaterite CaCO_3_ crystals via employing the fluidic self-assembly deposition method (Figure 15I) [53]. For the construction of the 3D porous scaffold, the CaCO_3_ crystals were packed inside a microfluidic channel (Figure 15(Ia)) and five alternate bilayers of PSS and PDADMAC were deposited on the cores (Figure 15(Ib)), followed by the removal of the CaCO_3_ using EDTA, leading to formation of hollow closely-packed PEMCs—i.e., a scaffold (Figure 15(Ic,Id)). The formed scaffold can be removed from the microchannel and will keep the dimensions of the channel in which it was formed, providing simple control of scaffold dimensions (Figure 15(Id–If)). The scaffold was analysed using SEM, indicating that the PEMCs were interconnected, as illustrated in Figure 15II. Cells can adhere to the porous PDADMAC/PSS scaffolds, and prefer scaffolds with the size of PEMC similar or comparable to the size of a cell (ca. 10 µm) [53]. Scaffolds formed with smaller or larger PEMCs have been shown to be cell repellent, and this may be caused by both topological or mechanical issues due to scaffold internal structure. One would expect that such scaffolds assembled from biopolymers such as the hyaluronic acid/PLL pair may also possess good cell adhesion if their mechanical properties are suitable [122].

## 6. Conclusions and Future Perspectives

Scaffolds formed using PEMCs templated onto vaterite crystals are becoming increasingly popular in the field of TE. This is due to the versatile approaches for encapsulation into such PEMCs; performed at fully biocompatible conditions, reaching a large internal concentration of bioactives, as is desirable for long-term release. Cargo release from PEMCs can be tailored to specific bioapplications, providing the burst or sustained release required for scaffolds utilised within TE applications, and may be induced by a variety of stimuli. PEMCs can be integrated either by incorporation into the polymer matrix of the scaffold, or the scaffold can be constructed fully of the PEMCs themselves. In the latter case, the porosity of the scaffolds can be controlled via the packing and size of the PEMCs. The porosity in such PEMC-based scaffolds is crucial for cell adhesion and bioactive retention. So far, the literature findings show that there is a set of tools to effectively adjust the porosity.

One has to pay attention to defects within the multilayer structure and osmotic pressure effects resulting from the fabrication process of such PEMCs. This excessive pressure may affect PEMC integrity and properties. The use of vaterite as a decomposable template reduced the risk of the enhanced osmotic pressure effects (due to small size of ions and their quick and complete release) and therefore minimise the chance of a defective multilayer structure. At the same time, the core decomposition should be slow to keep the ionic strength close to physiologically relevant value or below.

We strongly believe that, in the near future, the assembly of PEMC-based scaffolds using active PEMCs able to release bioactives on demand upon the introduction of external stimuli will significantly contribute to the new generation of biomaterials for TE and regenerative medicine. The integration of soft temperature-sensitive components into the multilayers will open new avenues to assemble externally activatable PEMCs fully constructed of polymers [224] or those of a hybrid nature having metal NPs [225]. This is the future trend and will aim at the ultimate goal of assembling systems mimicking the ECM and holding remote control over ECM properties. We would suggest that, in the future, the use of naturally derived components—first of all, nano-sized, but also micro-sized—will be a logical step for the assembly of such active scaffolds. This is driven by the strong demand from the industrial applications for low-cost materials. Examples of such materials are halloysites, vaterites and nanocellulose; the use of these materials for scaffold formulation in the future is highlighted elsewhere [226].
